# Factors Influencing User Engagement of Health Information Disseminated by Chinese Provincial Centers for Disease Control and Prevention on WeChat: Observational Study

**DOI:** 10.2196/12245

**Published:** 2019-06-27

**Authors:** Yan Zhang, Tingsong Xia, Lingfeng Huang, Mingjuan Yin, Mingwei Sun, Jingxiao Huang, Yu Ni, Jindong Ni

**Affiliations:** 1 Guangdong Medical University Dongguan China; 2 Baoan District Shajing Health Supervision Office Shenzhen China; 3 Beijing Jiaotong University Beijing China

**Keywords:** WeChat, WeChat official accounts, user engagement, CDC, health education

## Abstract

**Background:**

Social media is currently becoming a new channel for information acquisition and exchange. In China, with the growing popularity of WeChat and WeChat official accounts (WOAs), health promotion agencies have an opportunity to use them for successful information distribution and diffusion online.

**Objective:**

We aimed to identify features of articles pushed by WOAs of Chinese provincial Centers for Disease Control and Prevention (CDC) that are associated with user engagement.

**Methods:**

We searched and subscribed to 28 WOAs of provincial CDCs. Data for this study consisted of WeChat articles on these WOAs between January 1, 2017 and December 31, 2017. We developed a features frame containing title type, article content, article type, communication skills, number of marketing elements, and article length for each article and coded the data quantitatively using a coding scheme that assigned numeric values to article features. We examined the descriptive characteristics of articles for every WOA and generated descriptive statistics for six article features. The amount of reading and liking was converted into the level of reading and liking by the 75% position. Two-category univariate logistic regression and multivariable logistic regression were conducted to explore associations between the features of the articles and user engagement, operationalized as reading level and liking level.

**Results:**

All provincial CDC WOAs provided a total of 5976 articles in 2017. Shanghai CDC articles attracted the most user engagement, and Ningxia CDC articles attracted the least. For all articles, the median reading was 551.5 and the median liking was 10. Multivariable logistic regression analysis revealed that article content, article type, communication skills, number of marketing elements, and article length were associated with reading level and liking level. However, title type was only associated with liking level.

**Conclusions:**

How social media can be used to best achieve health information dissemination and public health outcomes is a topic of much discussion and study in the public health community. Given the lack of related studies based on WeChat or official accounts, we conducted this study and found that article content, article type, communication skills, number of marketing elements, article length, and title type were associated with user engagement. Our study may provide public health and community leaders with insight into the diffusion of important health topics of concern.

## Introduction

Acquisition and dissemination of health information play a significant role in promoting positive health behavior change [1]. Social media is currently becoming a new channel for information acquisition and exchange [2,3]. Nearly one-third of the world’s population uses social media for entertainment, study, work, and socializing [4]. Compared with traditional types of print and broadcast media, social media has a unique advantage in facilitating two-way communication, allowing organizations to personalize content and interact with the public [5]. With the popularity of social media in the public, the use of these tools by health education organizations will have many opportunities to influence and change health behaviors [6].

WeChat, a free mobile app released in 2011, has become the most widely and frequently used social media platform in China [2]. WeChat has many functions, including instant messaging, free phone calls, mobile payments, etc [7]. In addition, a new functional module of WeChat called WeChat official accounts (WOAs) can be freely used by governments, companies, and organizations to provide information called pushed articles to the public; individuals can freely read these articles and communicate with others via these official accounts [3,8]. According to the latest data, the number of monthly active users of WeChat has reached 650 million and the number of WOAs exceeds 10 million. Nearly 80% of WeChat users have subscribed to the WOA [9]. Studies have found that WeChat can successfully encourage health improvement and behavior change [10]. For example, Wei et al [3] used WOAs to improve malaria health literacy. Cao et al [8] found that giving additional education and instruction via WOAs can improve therapy outcomes of patients. A weight loss intervention campaign based on a WOA was found to be effective for males [10]. With the growing popularity of WeChat and WOA in health knowledge acquisition, their roles in health education and health intervention are gradually receiving more attention [11].

The widespread public engagement with WeChat creates a ready platform for health promotion agencies for successful information distribution and diffusion online [12]. The China Centers for Disease Control and Prevention (CDC) are professional institutions that conduct health education and health promotion work for the public. In order to broaden health communication and make it easier for users to obtain information, the China CDC opened the Chinese disease control dynamics WOA in April 2014 [13]. Most of the provincial CDCs have now opened WOAs [14]. Generating user engagement is vital for effective information diffusion and health promotion. Identifying predictors of social media engagement can guide the development of content and use of features that have high appeal for the public [15]. User engagement was defined as users reacting to (ie, reading, liking) any content [16,17]. Past research has identified strategies for successful user engagement on Facebook and Twitter, including using multimedia content, highlighting celebrity involvement, using humor or shock appeals, etc [17,18]. However, there is little evidence establishing the best ways to engage with the public using WeChat, highlighting the importance of further exploration of this area. The literature base exploring user engagement through the WOAs of CDCs is even more limited. CDCs have the potential to enable broad dissemination of health information and messaging online and promote healthy behaviors, contributing to the development of social health [5].

In this study, we reviewed the use of WOAs by Chinese provincial CDCs. The study aim was to identify features of their articles that are associated with user engagement, which we defined as the level of reading and liking. Ultimately, we sought to formulate predictors of user engagement that would inform health education of public health organizations, so they can make use of WOAs to engage their target market and increase the effectiveness of changing health-related behaviors.

## Methods

### Data Source

We used the mobile WeChat app to search the official accounts by the name of the province and the key words “Centers for Disease Control and Prevention” and “CDC.” We found 28 WOAs of provincial CDCs on January 15, 2018, and subscribed to them. Data for this study consisted of WeChat articles found on these WOAs between January 1, 2017, and December 31, 2017.

### Main Indicators and Article Features Frame

For each article, we recorded the code of official accounts, push time, and amounts of article reading and liking. We developed a features frame for each article, referring to the research of Kite et al [17]. Specifically, we added article content, title type, and article length, which are features that may affect the effectiveness of the WOAs. Next, we conducted a presurvey on 100 articles. Based on the results of the survey, we have merged some of the less frequently used categories into other and made some modifications during iterative testing to make the features frame more relevant to public health communication. The final features framework with definitions is shown in Table 1. Then we invited six experts in relevant fields to evaluate the content validity of the features frame and calculated the content validity index (CVI). Experts used a 4-point Likert scale to assess the degree of consistency between the content of each item and the corresponding article features. Very unrelated was counted as 1 point, comparatively unrelated was counted as 2 points, comparatively related was counted as 3 points, and very relevant was counted as 4 points. The CVI of the item level was 0.83 to 1.00, and the CVI of the entire framework was 0.98.

**Table 1:** Final features frame with definitions.

Item		Definition
**Title type**		
	Declarative sentence	The sentence contains subject, predicate, and object, usually with a period, aiming to state a fact. Example: The Centers for Disease Control and Prevention organized a series of women’s day activities.
	Exclamation or emphatic sentence	The sentence contains the subject, predicate, and object with an exclamation point. Example: These things will affect all doctors!
	Question sentence	The sentence contains the subject, predicate, and object with a question mark. Example: Can the leftovers be eaten?
	Imperative sentence	The sentence contains predicate and object, without subject, with an exclamation point. Example: Beware of these invisible drugs!
	Phrase	Not a complete sentence, no predicate. Example: Common cold and flu.
	Other	The title contains two or more sentences that are belong to different types. Example: Forget your flu shot? It is never too late!
**Article content**		
	Infectious disease	Topic is related to infectious diseases. Example: Deciphering new knowledge about AIDS.
	Chronic diseases	Topic is related to chronic diseases. Example: Women are more likely to develop diabetes. How can we prevent this?
	Food safety and nutrition	Topic is related to food safety or nutrition. For example, the topic introduces food effects and gives recommendations.
	Vaccination	Topic is related to vaccination, such as the introduction and guidance of some vaccines. Example: Forget your flu shot? It is never too late!
	Environmental and occupational health	Topic is related to air hygiene, drinking water hygiene, soil hygiene, housing hygiene, occupational diseases, etc. Example: How air pollution affects the view.
	Health education activities	Topic is related to events organized to conduct health education. Example: AIDS Day campaign.
	Healthy lifestyle	Topic is related to popular knowledge of life, such as how to lose weight, the benefits of drinking water, the dangers of staying up late, etc.
	Research progress	Topic is related to scientific research projects, including the laboratory construction of institutions.
	National health policy, conferences, etc	Topic is related to national meetings and policies related to the health industry, such as the National Health and Wellness Conference.
	Other	Content of the article is related to the unit meetings, work arrangements, introduction of advanced deeds, etc.
**Article type**		
	Text onl**y**	Article contains only text.
	Text and pictures	Article contains pictures and text.
	Text, pictures, and links	Article contains pictures, links, and text.
	Text, pictures, and videos	Article contains pictures, videos, and text.
	Other	Other types or other combinations.
**Communication skills**		
	Informative/instructive	Provides information on a health issue or instruction on how to do a behavior, such as health-related behavioral guidance.
	Questioning	Remains skeptical and professionally corrects knowledge about widely circulated opinions, remarks or certain articles. Example: No rumors! True facts about dietary supplements.
	Positive emotional appeal	Aims to elicit positive emotions, such as using positive examples to convey hope and excitement.
	Fear appeal	Uses negative cases to cause user fear or other negative emotions, such as discussing serious consequences of long-term smoking, etc.
	Humor	Uses any humorous technique (such as sarcastic, jokes, etc) to convey health messages, including funny pictures.
	Other	Uses two and more of the skills mentioned above.
**Marketing elements**		
	Persons of authority	Any person used for the purpose of lending their personal or positional authority to the health issue (eg, doctor, academic, scientist, politician).
	Celebrities and sports people	Connecting people related to entertainment media or sports profiles with health events.
	Information sources	Citing sources of information such as books, guides, references, or instructions from other platforms.
	Competitions, prizes, or giveaways	Any contest involving a participant entry, including minimal requirements such as liking or commenting on a article.
	Hot topics	Using internet or real-life hot topics such as news coverage.
	Tests	Collecting or testing participants’ health knowledge.
	Votes	Involving various types of voting for events or selections.
**Article length**		
	0-1000 words	Amount of words in the article text.
	1000-1499 words	Amount of words in the article text.
	1500-1999 words	Amount of words in the article text.
	2000 or more words	Amount of words in the article text.

### Coding Method

We coded the data quantitatively using a coding scheme that assigned numeric values to article features. Title type, article content, article type, communication skills, and article length contained mutually exclusive categories, and they were coded with the corresponding number. However, multiple marketing elements are possible in a single article, so not all categories were mutually exclusive. Each marketing element was coded as 0 or 1 (0 represents not using, 1 represents using). Next we tested the interrater reliability between the two coders. After receiving training and getting familiar with the content, LFH and MJY independently coded the same subset of articles (n=300) from 28 WOAs. Any disagreement between the coders was resolved by discussion. Once interrater reliability reached 80%, LFH and MJY then individually coded half of the WOAs each.

### Statistical Analyses

For every WOA, we examined the descriptive characteristics of articles, including the number and percentage of articles pushed in a year, median reading, and median liking per article. Next we generated descriptive statistics for each title type, article content, article type, communication skill, marketing element, and article length. We then investigated associations between the features of the articles and user engagement, operationalized as reading and liking. Since the data distribution is skewed, we categorized the amount of reading and liking by the 75% position, defining an amount of reading and liking less than the 75% position as low-level reading and liking and above the 75% position as high-level reading and liking. Given that the marketing elements were multichoice, we converted the features of marketing elements into the number of marketing elements. Next, we applied logistic regression analyses to assess associations between the features of the articles and the user engagement with the level of reading and the level of liking as the outcome variables and six features as categorical independent variables and used the *P* value to represent the result of the hypothesis test, which decided whether to reject the null hypothesis (the regression coefficient is equal to 0). We conducted a series of two-category univariate logistic regression analyses to perform the initial screening of variables. Variables that gave *P*<.10 in the univariate analyses were evaluated further using multivariable logistic regression. For the multivariable regression, *P*<.05 was considered the statistically significant level. Risks were expressed as adjusted odd ratios with 95% confidence intervals. EpiData 3.1 software (EpiData Association) was used to establish the database; SPSS Statistics version 15.0 (IBM Corp) was used for the statistical analysis.

## Results

### Characteristics of WeChat Official Accounts and Article Features

Overall, 28 provincial CDC WOAs pushed a total of 5976 articles in 2017 (Table 2). Guangdong CDC pushed the most articles (565/5976, 9.45%), and Hainan CDC pushed the fewest articles (4/5976, 0.07%). Shanghai CDC articles attracted the most user engagement (median reading: 3777, median liking: 37), and Ningxia CDC articles attracted the least (median reading: 21, median liking: 0). For all articles, the median reading was 551.5 and the median liking was 10.

As described in Table 3, article titles were usually declarative sentences (2358/5976, 39.45%). Most articles were related to healthy lifestyle (1355/5976, 22.67%), and text with pictures (3686/5976, 61.68%) was the most common article type. The most common communication skill was informative or instructive (3096/5976, 51.81%). Only 60.66% (3625/5976) of the articles contained any marketing elements, and the most articles used only one kind of marketing element (2543/5976, 42.55%). The article length was usually 1000 words or fewer (3615/5976, 60.49%).

**Table 2:** Characteristics of the 28 included WeChat official accounts (N=5976).

Province of the WeChat official channel	Articles, n (%)	Median reading	Median liking
Zhejiang	31 (0.5)	409	9
Fujian	205 (3.4)	281	9
Guangdong	565 (9.5)	1715	20
Hunan	210 (3.5)	2239	19.5
Hubei	529 (8.9)	956	16
Hainan	4 (0.1)	49.5	1.5
Yunnan	475 (7.9)	413	11
Guizhou	328 (5.5)	205.5	4
Sichuan	78 (1.3)	418.5	13
Qinghai	160 (2.7)	34	0
Gansu	185 (3.1)	270	4
Shanxi	231 (3.9)	1568	13
Jilin	223 (3.7)	163	3
Liaoning	358 (6.0)	211.5	3
Hebei	321 (5.4)	197	2
Jiangxi	154 (2.6)	200.5	8
Jiangsu	445 (7.4)	2498	31
Anhui	66 (1.1)	791	17
Henan	82 (1.4)	226.5	4
Shandong	211 (3.5)	393	9
Shanxi	55 (0.9)	127	4
Beijing	355 (5.9)	750	12
Shanghai	323 (5.4)	3777	37
Chongqing	59 (1.0)	525	23
Xinjiang	20 (0.3)	429	15
Guangxi	19 (0.3)	80	2
Ningxia	165 (2.8)	21	0
Xizang	119 (2.0)	180	2

**Table 3:** Frequencies by category of six article features (n=5976).

Article features		Value, n (%)
**Title type**		
	Declarative sentence	2358 (39.45)
	Exclamation or emphatic sentence	1011 (16.92)
	Question sentences	858 (14.36)
	Imperative sentence	517 (8.65)
	Phrase	704 (11.78)
	Other	528 (8.84)
**Article content**		
	Infectious disease	910 (15.23)
	Chronic diseases	385 (6.44)
	Food safety and nutrition	578 (9.67)
	Vaccination	350 (5.86)
	Environmental and occupational health	221 (3.70)
	Health education activities	625 (10.46)
	Healthy lifestyle	1355 (22.67)
	Research progress	89 (1.49)
	National health policy, conferences, etc	421 (7.04)
	Other	1042 (17.44)
**Article type**		
	Text only	264 (4.42)
	Text and pictures	3686 (61.68)
	Text, pictures, and links	1576 (26.37)
	Text, pictures, and videos	268 (4.48)
	Other	182 (3.05)
**Communicative skills**		
	Informative/instructive	3096 (51.81)
	Questioning	314 (5.25)
	Positive emotional appeal	1019 (17.05)
	Fear appeal	704 (11.78)
	Humor	258 (4.32)
	Other	585 (9.79)
**Number of marketing elements**		
	None	2351 (39.34)
	One	2543 (42.55)
	Two	910 (15.23)
	Three or more	172 (2.88)
**Article length**		
	0-1000 words	3615 (60.49)
	1000-1499 words	1573 (26.32)
	1500-1999 words	449 (7.51)
	2000 or more words	339 (5.67)

### Association Between Article Features and Level of Reading and Liking

As shown in Table 4, univariate logistic regression analysis revealed that title type, article content, article type, communication skills, number of marketing elements, and article length were significantly related to reading level (*P*<.001). Liking level displayed a similar pattern. All article features were evaluated further using multivariable logistic regression.

Articles for which the title type was an exclamation or emphatic sentence were less likely to obtain a high level of liking than those whose title types were declarative sentences. With regard to article content, articles about infectious diseases, vaccination, food safety and nutrition, and healthy lifestyle were more likely to receive high-level reading and liking than those about other topics. Articles related to health education activities or national health policy conferences were less likely to obtain high-level reading and liking. Environmental and occupational health–related articles were more likely to obtain high-level reading, unlike articles describing research progress, but both showed no effect for level of liking. For article type, articles containing text, pictures, and links were 5.21 times and 5.64 times more likely to receive high-level reading and liking, respectively, than text-only articles. Those containing text and pictures, similar to those containing text, pictures, and videos, were also more likely to receive high-level reading. With regard to communication skills, when compared with informative or instructive articles, those using traits of humor, questioning, fear appeal, and other were more likely to receive high-level reading and liking. Articles with positive emotional appeal were more likely to obtain a high-level liking, but this had no effect observed for level of reading. Articles using three or more kinds of marketing elements were more likely to obtain high-level reading and liking than those using none, while articles using one and two kinds were both less likely to obtain high levels of reading and liking. Compared with articles containing 1000 words or fewer, those containing 1000 to 1500 words and 1500 to 2000 words were more likely to obtain high-level reading and liking.

**Table 4:** Univariate and multivariable logistic regression analysis.

Article features		Univariate logistic regression		Multivariable logistic regression			
		Reading level, *P* value	Liking level, *P* value	Reading level		Liking level	
				Odds ratio (95% CI)	*P* value	Odds ratio (95% CI)	*P* value
**Title type**		<.001	<.001	—	—	—	—
	Declarative sentence (reference)	—	—	1	—	1	—
	Exclamation or emphatic sentence	.18	.60	—	—	0.80 (0.65-0.97)	.02
	Question sentence	<.001	<.001	—	—	—	—
	Imperative sentence	.04	.14	—	—	—	—
	Phrase	.26	.22	—	—	0.69 (0.55-0.87)	.001
	Other	<.001	<.001	—	—	—	—
**Article content**		<.001	<.001	—	—	—	—
	Infectious disease	<.001	<.001	3.33 (2.60-4.25)	<.001	2.15 (1.69-2.73)	<.001
	Chronic diseases	.57	.05	—	—	—	—
	Food safety and nutrition	<.001	<.001	1.76 (1.34-2.31)	<.001	1.54 (1.18-2.01)	.001
	Vaccination	<.001	.14	2.93 (2.15-4.00)	<.001	1.76 (1.29-2.41)	<.001
	Environmental and occupational health	.48	.85	1.54 (1.05-2.26)	.03	—	—
	Health education activities	<.001	<.001	0.65 (0.48-0.88)	.01	0.64 (0.48-0.86)	.002
	Healthy lifestyle	<.001	<.001	1.89 (1.50-2.38)	<.001	1.39 (1.11-1.74)	.004
	Research progress	<.001	.01	0.20 (0.05-0.82)	.03	—	—
	National health policy, conferences, etc	<.001	<.001	0.30 (0.18-0.50)	<.001	0.38 (0.25-0.59)	<.001
	Other (reference)	—	—	1	—	1	—
**Article type**		<.001	<.001	—	—	—	—
	Text only (reference)	—	—	1	—	1	—
	Text and pictures	.02	<.001	1.62 (1.09-2.40)	.02	2.31 (1.48-3.62)	<.001
	Text, pictures, and links	<.001	<.001	6.21 (4.12-9.35)	<.001	6.64 (4.20-10.48)	<.001
	Text, pictures, and videos	<.001	<.001	2.24 (1.36-3.69)	.002	2.60 (1.51-4.47)	.001
	Other	.32	.42	—	—	—	—
**Communication skills**		<.001	<.001	—	—	—	—
	Informative/instructive (reference)	—	—	1	—	1	—
	Questioning	<.001	<.001	1.83 (1.40-2.39)	<.001	2.05 (1.57-2.67)	<.001
	Positive emotional appeal	.01	.03	—	—	1.22 (1.00-1.49)	.048
	Fear appeal	<.001	<.001	1.34 (1.10-1.64)	.004	1.44 (1.18-1.77)	<.001
	Humor	<.001	<.001	2.93 (2.20-3.90)	<.001	4.13 (3.11-5.49)	<.001
	Other	<.001	<.001	1.59 (1.25-2.02)	<.001	1.55 (1.22-1.97)	<.001
**Number of marketing elements**		<.001	<.001	—	—	—	—
	None (reference)	—	—	1	—	1	—
	One	.03	<.001	0.83 (0.72-0.96)	.01	0.76 (0.66-0.88)	<.001
	Two	.01	<.001	0.62 (0.50-0.76)	<.001	0.60 (0.49-0.74)	<.001
	Three or more	<.001	<.001	1.63 (1.13-2.36)	.01	1.86 (1.30-2.67)	.001
**Article length**		<.001	<.001	—	—	—	—
	0-1000 words	—	—	1	—	1	—
	1000-1499 words	<.001	<.001	1.45 (1.25-1.68)	<.001	1.42 (1.23-1.65)	<.001
	1500-1999 words	<.001	<.001	1.67 (1.32-2.11)	<.001	1.55 (1.22-1.96)	<.001
	2000 or more words	.97	.78	—	—	—	—

## Discussion

### Principal Findings

To our knowledge, this is the first study to investigate the current status of WOAs of CDCs for health information dissemination. Our analysis identified several features of articles that were associated with better information dissemination, which we defined as high-level user engagement. In the field of public health research and practice, WeChat represents a convenient and accessible tool for health education in China, which historically has been difficult using traditional methods [2,19]. Health promotion organizations should be aware of strategies to engage their target audience. The results presented in this paper may provide these organizations with some guidance on how to improve engagement with WeChat users.

According to our results, the content of articles was associated with user engagement. Indeed, article content was identified as an essential factor in determining whether WeChat users forward or share articles with friends [20]. Research on other social media platforms has also revealed that the content of posts seems to have a significant effect on user engagement [16,21]. Our findings further showed that what the public liked to read and praise were articles about infectious diseases; vaccination; food safety and nutrition; and healthy lifestyles, which reflects that they seem to be interested in popular science articles about daily health knowledge. In contrast, the public was relatively less interested in articles related to research progress and health education activities of institutions or national health policy, which may seem to be far away from daily life.

As the results showed, article type was an important covariate of user engagement. This is consistent with previous research. Research found that article type is an important indicator that alters the effectiveness of article dissemination on WOAs [22]. Among studies based on Facebook, the effect of the post type is also displayed. One found higher engagement to be associated with pictures, videos, and links [16]. Another showed that video posts were more attractive post types than picture posts [17]. Our study seems to give another possibility related to the combination of article types. A combination of text, pictures, and links was the most engaging article type, 5 times more popular than text only. Although the combination of text, pictures, and videos and the combination of text and pictures were also more likely to get a high-level reading and liking, the effect was far lower. This seems to indicate that links play a more important role in the combination of article types for increasing the user engagement. The effect of the links can be easily found in the advertising field. Ads may work better if the users can directly access the relevant pages through the links in the social media website pages instead of being forced to visit the external website [23]. And in the field of health promotion using networking platforms, relevant links are also identified as a key strategy for successful user engagement [24]. However, only 26.4% of all articles we coded included the combination of text, pictures, and links and the combination of text and pictures accounted for 61.7%, which suggested that public health organizations were trailing behind marketers in advertising field. The administrators of the CDC official accounts should probably add links to the traditional combination of text and pictures.

The communication skills used were also related to user engagement. Compared with the use of a peaceful tone to convey health knowledge, articles using humor were more likely to receive high levels of reading and liking. This result is similar to the findings of previous research: Klassen et al [25] found that using an optimistic tone was associated with more interactions on Facebook, and posts from health promotion organizations that were more serious in tone had minimal engagement from fans. This may reflect the effect of emotions [26]. Some researchers think that emotion is an important motivator of social sharing, and when participants experience positive emotions viewing a post on social media, they are more likely to engage with that post than are those who do not experience positive emotions [27,28]. In addition, articles using questioning skills were also nearly two times more likely to achieve high-level reading and liking than those using informative or instructive skills. Some surveys have found that many respondents believe that internet health information is not reliable [2,29]. Indeed, there are potential dangers associated with using social media for health communication, such as sharing of misleading or inaccurate information [5]. As professional health organizations, CDCs use questioning expressions to correct this information, which may attract readers’ attention. According to our results, articles with fear appeal were likely to show similar but slightly lower results than those using the above two skills. In the past, fear has been employed by many health promotion organizations to induce behavior change [25]; however, Soames Job [30] and Keller [31] think that fear is only likely to work under particular circumstances. Therefore, the effectiveness of fear appeal may require further research.

The number of marketing elements was associated with the level of user engagement. Articles that used one or two marketing elements were less likely to obtain high-level reading and liking than those that used none. However, articles using three or more kinds of element obtained the opposite result. This showed that some marketing elements were associated with lower levels of engagement and some with higher engagement. Previous research has discussed the impact of some marketing elements on user engagement in other social media. The effect of celebrities is usually positive. Chapman [32] believed that celebrity involvement in public health campaigns delivers long-term benefit. Similarly, Kite [17] found that the use of celebrities and athletes led to higher average engagement. However, the effect of authoritative people is still controversial. One study found that, compared with Facebook posts that have no marketing elements at all, using authoritative people reduced likes, shares, and comments [17]. But Preece et al [21] thought that using charismatic leaders or respected authorities increased the chances of user engagement. Therefore, which marketing methods can play positive roles in increasing the user engagement on articles of WOAs remains to be further discussed.

The results of this study showed that article length was also a covariate of user engagement: articles containing 1000 to 1500 words and 1500 to 2000 words were more likely to obtain high-level reading and liking. A study of WeChat articles considered that text above 1500 words can be considered as a long text [33]. Our study showed that users seem to have a preference for articles of moderate length.

In our study, title type was negatively associated with user engagement. When the title was an exclamation or an emphatic sentence or phrase, the articles were less likely to receive high-level liking compared with titles involving declarative sentences. Zhang [34] analyzed the influence of WeChat article titles on people’s willingness to open the full text and found that the content of the title could affect the dissemination of the article. However, we do not have sufficient evidence to demonstrate what type of title was more popular with readers. Therefore, the link between title type and user engagement seems to present an interesting direction for future research.

### Limitations

Our findings should be interpreted with consideration of some study limitations. First, the study involved a short time frame of data collection. Evaluating a longer period may identify time differences and improve strategies. Second, this study used reading and liking as indicators of user engagement, and we further transformed them into two-category variables: high-level and low-level. We may thus be obscuring the effects of the original variables. Future analyses should expand our findings by quantitatively evaluating the indicators associated with engagement. Last, other factors that we have not focused on in this study may also shape user engagement such as individual-level factors. These individual factors and article features should be analyzed comprehensively in future research.

### Conclusions

Social media involves technologies that facilitate opportunities for engaging with the audience [35]. How social media can best be used to achieve health information dissemination and public health outcomes is a topic of much discussion and study in the public health community. Given the lack of related studies based on WeChat or official accounts, we conducted this study and found that the article content, article type, communication skills, the number of marketing elements, and article length were associated with reading level and liking level. However, title type was only associated with liking level. Our results may provide public health and community leaders with insight into the diffusion of important health topics of concern. Because this study was focused primarily on user engagement and article features, future studies might improve our understanding of other factors that contribute to the dissemination of specific key themes. For example, the agency must identify what audience they are trying to reach, how that audience uses WeChat, and what goals and objectives are most appropriate.

## Abbreviations

CDCs: Centers for Disease Control and Prevention
CVI: content validity index
WOA: WeChat official account
